# New Insights into the Process of Placentation and the Role of Oxidative Uterine Microenvironment

**DOI:** 10.1155/2019/9174521

**Published:** 2019-06-25

**Authors:** Sara Mendes, Filipa Timóteo-Ferreira, Henrique Almeida, Elisabete Silva

**Affiliations:** ^1^Ageing and Stress Group, IBMC (Instituto de Biologia Molecular e Celular), I3S (Instituto de Investigação e Inovação em Saúde), Universidade do Porto, Rua Alfredo Allen 208, 4200-135 Porto, Portugal; ^2^Unidade de Biologia Experimental, Departamento de Biomedicina, Faculdade de Medicina, Universidade do Porto, Alameda Professor Hernâni Monteiro, 4200-319 Porto, Portugal; ^3^Ginecologia-Obstetrícia, Hospital-CUF Porto, Estrada da Circunvalação 14341, 4100 180 Porto, Portugal

## Abstract

For a successful pregnancy to occur, a predecidualized receptive endometrium must be invaded by placental differentiated cells (extravillous trophoblast cells (EVTs)) and, at the same time, continue decidualization. EVT invasion is aimed at anchoring the placenta to the maternal uterus and ensuring local blood supply increase necessary to provide normal placental and foetal development. The first is achieved by migrating through the maternal endometrium and deeper into the myometrium, while the second by transforming uterine spiral arteries into large vessels. This process is a tightly regulated battle comprising interests of both the mother and the foetus. Invading EVTs are required to perform a scope of functions: move, adhere, proliferate, differentiate, interact, and digest the extracellular matrix (ECM); tolerate hypoxia; transform the maternal spiral arteries; and die by apoptosis. All these functions are modulated by their surrounding microenvironment: oxygen, soluble factors (e.g., cytokines, growth factors, and hormones), ECM proteins, and reactive oxygen species. A deeper comprehension of oxidative uterine microenvironment contribution to trophoblast function will be addressed in this review.

## 1. Introduction

Successful pregnancy depends on sequential and discrete events that include fertilization, implantation, decidualization, placentation, and birth. Placentation is the process of formation and development of the placenta and the associated modifications in maternal tissue. Its continued interaction character, involving two distinct genomes, suggests the presence of a fine-tuned regulation. In human placenta development, three structural regions are considered: the foetal placenta, with separated foetal and maternal blood, where physiological exchange of nutrients and waste products occurs; the basal plate, which borders the maternal surface and is crossed by maternal vessels; and the placental bed formed by maternal uterine tissue, comprising the modified endometrium (decidua) and is traversed by 100-150 maternal spiral arteries that supply nutrients and oxygen (O_2_) to the placenta and the foetus [1].

For a healthy pregnancy to proceed, a good anchoring of placental features and the transformation of maternal spiral arteries (SA) into flaccid capacitation vessels, that will ensure adequate blood supply to the foetus, are necessary. In normal pregnancy, such changes require important extravillous trophoblast cell (EVT) movement from the placental villi across the decidua and deep into the adjacent myometrium. In addition, appropriate invasion of maternal SA and their resulting remodelling underlies functional circulatory change establishment [2]. In contrast, deficient EVT invasion has been associated with insufficient SA remodelling, altered uteroplacental hemodynamics, overall placenta bed dysfunction, and the establishment of serious pregnancy complications [3]. In fact, an early defective development of the placental bed, and consequent altered placentation, appears to contribute to late pregnancy complications such as preeclampsia, placental abruption, and intrauterine growth restriction (IUGR) [1, 2].

EVT invasion regulation and the molecular mechanisms underlying SA remodelling are the result of a complex network involving soluble factors and different cell types residing in the maternal placental bed. Emerging work indicates that an abnormal placentation is consequent to aberrant uterine microenvironment, already present before or at the time of blastocyst implantation [4–7]. This review will address uterine regulators of EVT dynamics with a special focus on reactive oxygen species (ROS) physiological and pathophysiological roles.

## 2. Pre(decidualization)

In many species, uterine changes aiming to create a suitable microenvironment for embryo implantation and development occur only after implantation. In humans, early changes may be recognized after ovulation and are designated predecidualization [8]. In the uterine stroma adjacent to SA, and in response to rising progesterone levels, fibroblast-like mesenchymal cells differentiate into an epithelioid structure. In addition, they accumulate cytoplasmic glycogen and lipids and secrete new products as components of extracellular matrix (ECM), protease inhibitors, cytokines, hormones, and other peptides. If implantation takes place, they will provide nutrition to the developing conceptus [9].

Progesterone-dependent differentiation of stroma cells is crucial for epithelium receptiveness and trophoblast-endometrium interactions. In fact, trophoblast spheroid attachment and growth in a coculture of endometrial epithelial cells and primary stromal cells were increased when stromal cells had been collected during the window of implantation time, not before [10].

Predecidualization also plays an important role in uterine natural killer (uNK) cell influx. In humans, they are recruited during predecidualization, and their increase peaks during the first trimester and diminishes thereafter, due to apoptosis. When compared with circulating NK cells, uNK cells have distinct features and functions. They are less cytotoxic and produce signalling molecules such as cytokines (e.g., tumour necrosis factor alpha (TNF-*α*) and interleukin- (IL-) 10 and 1*β*), growth factors (e.g., tissue growth factor beta (TGF-*β*) and placental growth factor (PlGF)), angiogenic factors (e.g., vascular endothelial growth factor (VEGF)), and matrix metalloproteinases (MMPs) [11]. Moreover, they contribute to decidual angiogenesis regulation and SA remodelling and control EVT invasion [12].

## 3. Implantation and Early Placentation

**Figure 1:**
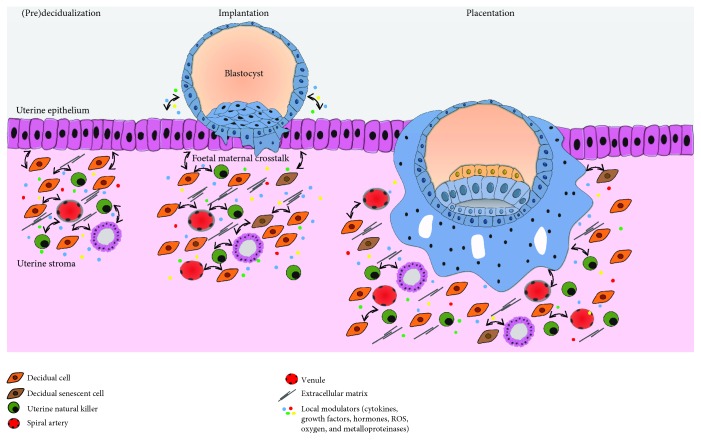
Human placenta development. Blastocyst implantation is mediated by the crosstalk between the blastocyst and the receiving endometrium. Early differentiated syncytiotrophoblast, displaying an invasive phenotype, allows the blastocyst to implant inside the endometrial stroma. Cytokines, growth factors, hormones, oxygen, extracellular matrix, and ROS all modulate trophoblast cell invasion of maternal decidua and myometrium and their capacity to transform spiral arteries. Many growth factors and cytokines, such as EGF, TGF-*β*, and TNF-*α*, secreted by the decidua and uNK cells act in a paracrine manner to regulate trophoblast function. These factors may also be secreted by the trophoblast cells and act in an autocrine manner to promote invasion.

Upon fertilization, the ovum travels in the fallopian tube where following several mitotic divisions, it reaches the morula stage (a compact mass of 12-16 cells). Continuing to divide, while receiving nutrients from the uterine environment, it attains approximately 100 cells that surround a fluid-filled cavity, where conceptus-derived secretions concentrate, characterizing the blastocyst stage [13]. During this stage, asymmetric cell divisions give rise to two distinct populations: the outer blastocyst encircling trophoblast cells, which will originate both the placenta and the extraembryonic membranes, and the totipotent inner cell mass, which will develop into the embryo [14]. Between the 5^th^ and the 6^th^ day post fertilization, the blastocyst contacts with the uterine wall (apposition), attaches to the epithelium, and invades the receptive decidua to implant [15, 16] (Figure 1).

After implantation, trophoblasts that face directly the maternal tissue differentiate and fuse to form the syncytiotrophoblast, whereas those remaining behind, untouched by maternal tissue, do not fuse and are denominated cytotrophoblasts [17, 18] (Figure 1). They act as a rapidly dividing stem cell pool that feeds and fuses with the multinucleated syncytiotrophoblast and promotes its continuous growth. Soon, it will surround most of the blastocyst and, with an invasive phenotype, will penetrate deep into the uterine cavity lining. Within the syncytium, fluid-filled spaces coalesce and rearrange into lacunae, which are the primitive intervillous spaces in the placenta, where the maternal blood will circulate [19, 20].

While invasion evolves, columns of the syncytiotrophoblast masses establish a network around the lacunae to form trabeculae, very important for the remaining development of the villous tree. Subsequently, cytotrophoblast cells proliferate and invade through the trabeculae, until they reach their tips and contact with the decidua. Following their lateral spreading from the tips, they form a coating between the syncytiotrophoblast mantle and the maternal endometrium [21]. Therefore, at this stage, the blastocyst exhibits three different layers of trophoblastic covering: (1) the primary/early chorionic plate, which faces the embryo; (2) the lacunar system and trabeculae, which develops into the intervillous space and villous tree, respectively; and (3) the cytotrophoblastic shell or primitive basal plate, which contacts directly with the endometrium [21].

The cells from the cytotrophoblastic shell possess a distinct phenotype, as they exhibit a round outline structure and large amounts of glycogen. Those localized at the tips of villi differentiate into EVTs, leave the shell, and migrate across the endometrium, initiating the process of EVT invasion [22]. A batch of EVTs is responsible for SA remodelling: they disrupt the vascular smooth cell layer and replace the endothelium, converting muscular wall arteries into wide bore low-resistance vessels ensuring a local increase in blood supply, necessary to fulfil placenta requirements [23]. At the same time, these cells accumulate and plug the lumen of the transformed SA, obstructing blood cell circulation. Nevertheless, there is a plasma leak which results in a physiological gradient of O_2_ between the mother and the foetus, with extreme importance for organogenesis [2]. In a phenomenon named deep placentation, EVTs further invade the decidua and reach as far as the inner third of the myometrium.

## 4. Modulators of EVT Function and Associated Signalling Pathways

**Figure 2:**
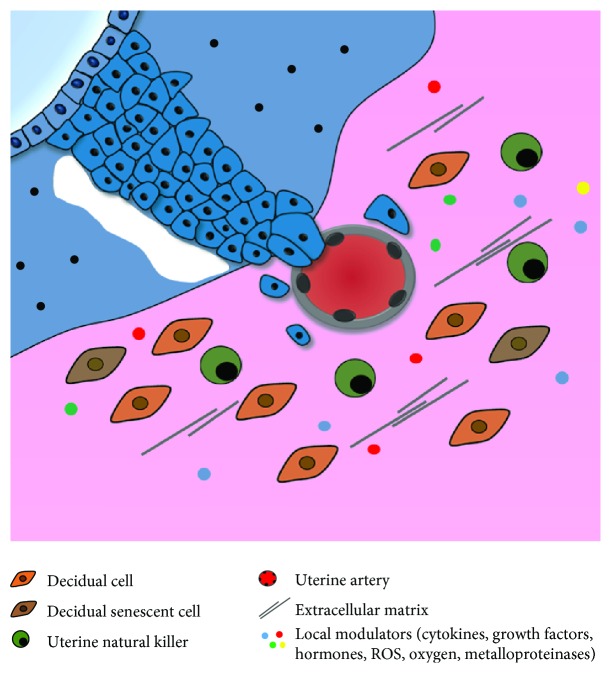
Extravillous trophoblast invasion and spiral artery remodelling. Within the syncytium, lacunae (the primitive intervillous space) are formed and proliferative cytotrophoblast cells emanate until they contact the endometrium (anchoring villi). At the tips of the villi, cytotrophoblasts differentiate into invasive trophoblast cells that will leave the villi and migrate through the stroma until they reach maternal spiral arteries or the deep myometrium. Interstitial extravillous trophoblasts that reach spiral arteries disrupt the vascular smooth muscle cell layer and replace it by fibrinoid material, while endovascular trophoblasts destroy their lumen and occupy their endometrium, converting them into low-resistance vessels.

Extravillous trophoblasts are not isolated elements as they are surrounded by decidual cells, vascular features, ECM proteins, uNK cells, and soluble factors, which together constitute the uterine microenvironment (Figure 2). This microenvironment must be suitable for an effective implantation that is the pillar for a successful pregnancy.

### 4.1. Oxygen

Low O_2_ levels are essential for correct placental development. In fact, during the first trimester of pregnancy, when SA are plugged by EVTs, there is an abrupt decrease in O_2_ concentration from the decidua to the developing placenta [24, 25]. This gradient is essential for cell column basement-residing cytotrophoblast cells to proliferate, reach the tips of the columns, and differentiate into invading extravillous trophoblasts. It thus appears that dividing cytotrophoblasts are pushed forward, towards maternal tissue and higher O_2_ levels, where they lose proliferative capacity, acquire an invasive phenotype, and start invading the maternal tissue [26]. Low O_2_ levels also induce the expression and stability of transcription factors, such as hypoxia-inducible factor-1 (HIF-1), which promotes expression of genes that encode proteins involved in cell metabolism, essential for trophoblast proliferation and differentiation [27].

### 4.2. Adhesion Molecules and Receptors

The transition from proliferating cytotrophoblast cells to invasive EVTs is also dependent on specific cell receptors and cell adhesion molecule (CAM) alterations. It starts with trophoblast cell detachment from the basal membrane and culminates with de novo adhesion to uterine ECM, enabling EVTs to further migrate and invade the myometrium and SA. A variety of molecules with a role in adhesion, motility, and migratory capacity are present in the EVTs and include integrins, selectins, cadherins, kisspeptins, and ephrins [28, 29]. Integrins are the major family of CAM with a key importance in the above-mentioned processes. Their expression differs among trophoblast populations and modulates the binding to the ECM. In addition, locally produced cytokines can influence CAM expression, particularly TGF-*β* [30]. EVT integrins bind to ECM proteins and other decidual molecules and activate cellular pathways controlling trophoblast functions [31].

### 4.3. Extracellular Matrix

The decidual ECM is a 3-dimensional tissue structure where trophoblast lineages are embedded. This matrix is composed of a variety of proteins including collagen, fibronectin, laminin, vitronectin, trophin, and tastin [32]. ECM modulate EVT functions and, at the same time, EVTs degrade and induce ECM remodelling to enable migration [33–35].

ECM proteins are degraded by proteases, cathepsins, and MMPs [36]. MMPs belong to the family of zinc-dependent endopeptidases, with diverse members that degrade distinct units of the ECM (Table 1) [37, 38].

Regulation of MMP expression can be done at different levels: transcriptional (e.g., cytokines and growth factors), during secretion, by extracellular activation (e.g., plasmin-activated MMP-3), by inhibition (e.g., tissue inhibitors of metalloproteinases (TIMPs)), or by degradation [59, 60]. TIMPs are a family of extracellular proteins (TIMP-1, TIMP-2, TIMP-3, and TIMP-4), which act as specific protease inhibitors, binding to the catalytic MMP domain and counteracting MMP activity [61].

Cell-matrix or cell-cell contact mediates both MMPs and TIMPs production [62]. To invade, EVTs must bind to ECM components, degrade them, and subsequently move through the tissue matrix. Cell surface adhesion molecules are essential for cell adhesion and constitutively express proteinases for ECM degradation [63]. Both EVT adhesion molecules and MMP secretion are dependent on ECM composition [36] and their phenotypic features. EVTs show an early predominant expression of MMP-2 that changes to MMP-9 later on during trophoblast invasion, to cope with decidual ECM alterations [64–67]. Overall, decidual cells, when in contact with EVTs, also express MMPs assisting in ECM degradation and further enhancing trophoblast invasion [67], but they also antagonize MMP activity by producing TIMPs and consequently blocking trophoblast invasion [68].

Decidual cells balance MMPs and TIMP secretion, control EVT migration, and prevent an exacerbate invasion [69] in a tight regulation and following a strict balance [70]. Thus, in order to achieve a correct placentation, uterine microarchitecture remodelling is necessary and requires a fine-tuned regulatory process operated by multiple players, of which only a limited number is currently known.

### 4.4. Soluble Factors—Cytokines and Growth Factors

Both timing and extension of EVT invasion are partly regulated by a plethora of paracrine and autocrine factors expressed by different cells comprising the decidua and EVTs themselves. Moreover, expression of these factors shows a considerable structural overlap, with several mediators being expressed by the decidua, uNK, and trophoblast cells [71]. In a decidualized endometrium, the cytokine/chemokine secretion is unique and, with the exception of leukaemia inhibitory factor (LIF), the expression of these soluble factors is increased when compared with nondecidualized stromal cells (Table 2).

Due to such alteration, it is conceivable that the decidual secretome has a role in controlling trophoblast invasion [73]. In a simplified way, soluble mediators can be divided in two groups: pro- and anti-invasive. Proinvasive paracrine factors, which have been shown to increase *in vitro* cell migration, invasion, and adhesion, comprise IL-1, IL-6, IL-8, IL-15, LIF, insulin-like growth factor-binding protein 1 (IGFBP-1), epidermal growth factor (EGF), interferon gamma-induced protein 10 (IP-10), RANTES (regulated on activation, normal T cell expressed and secreted), and chemokines CX3CL1 and CCL14. Anti-invasive factors include IL-10, IL-12, TNF-*α*, TGF-*β*, interferon gamma (IFN-*γ*), chemokine CXCL12, VEGF, and endocrine gland-derived VEGF (EG-VEGF) (Table 3).

Apart from the decidua, other tissues are producers of trophoblast regulators. Leptin, produced in the adipose tissue and in trophoblasts, can enhance EVT invasion capacity by an effective increase in MMP-14 expression [134–136]. In a placental bed, paracrine factors bind to the EVT cognate receptors and trigger signalling cascades that regulate gene expression and enzymatic activity, which induce a shift in MMPs, ILs, and growth factor secretion. This variation further regulates, in a feedforward fashion, a plethora of soluble factors that also control invasion.

### 4.5. Signalling Pathways

Several signalling pathways are responsible for controlling migration and invasion of EVTs including mitogen-activated protein kinase (MAPK), phosphoinositide 3-kinase (PI3K)/protein kinase B (Akt), Janus kinase (JAK)/signal transducer and activator of transcription proteins (STATs), wingless (Wnt), and focal adhesion kinase (FAK) pathways. However, endometrium-derived soluble factors predominantly activate MAPK, JAK/STAT, and TGF-*β*-mediated signalling pathways.

One of the most important pathways of MAPK signalling is extracellular signal-regulated kinase (ERK) 1/2. It participates in essential functions as cell proliferation, differentiation, and survival [137]. This pathway can be activated by mitogens, phorbol esters, growth factors, and ROS [137, 138]. In pregnancy, ERK1/2 is important for placental development [139], trophoblast differentiation, and decidual invasion [138, 140]. Endothelin and prostaglandins activate ERK1/2 and promote EVT migration, while inhibition of this pathway reduces it [140]. The p38 MAPK pathway is also an important MAPK signalling pathway; it is activated by cytokines [141], among other agents, and is necessary in the control of apoptosis, inflammation, cell cycle regulation, senescence, and oncogenesis [141, 142]. In particular, the p38*α* isoform plays a vital role in placental embryonic development and placental angiogenesis [143]. ERK1/2 inhibition in parallel with p38 MAPK decreases trophoblast differentiation [138]. Activation of the MAPK pathway in combination with the PI3K/Akt pathway promotes EVT (HTR-8/SVneo immortalized cell line) invasion and migration via MMP enhancement [144].

JAK/STAT3 signalling is indispensable for regulation of EVT proliferation and invasion capacity in response to cytokines and growth factors [145, 146]. Again, an interdependence between MAPK and JAK-STAT signalling pathways was found to be involved in EGF-mediated HTR-8/SVneo cell invasion [146].

TGF-*β* signals through Smad-dependent (canonical) and Smad-independent (ERK, JNK, p38, and Rho GTPases) (noncanonical) pathways. Recent studies with JEG trophoblast cells demonstrate that activation of Smad3 promotes cell invasion by upregulation of MMP2 and MMP9 [147]. These findings contrast with previous reports where TGF-*β* decreased EVT invasion in HTR-8/SVneo cells, by inducing Snail-mediated downregulation of vascular endothelial-cadherin [147]. TGF-*β* plays a role in multiple signalling networks in the cell, and depending on the second messengers involved, divergent responses can be attained.

ROS are important secondary messengers and play a role in the modulation of protein kinase activity. When a redox imbalance occurs, ROS can impair the EVT signalling network. Modification of essential amino acid residues by ROS, which consequently alter the protein structure and its function, is one of the plausible mechanisms of ROS actions [148].

## 5. Oxidative Stress and Placentation

### 5.1. Reactive Oxygen Species, Oxidative Stress, and Placentation

The ROS family comprises free radicals (i.e., species with at least one unpaired electron) and nonradical oxidants (i.e., oxidants with their electronic ground state complete). These species reactivity, half-lives, and diffusion capacities are variable. Hydroxyl radical (^·^OH) is the most unstable and upon formation reacts rapidly with biomolecules in the vicinity [149]. In contrast, hydrogen peroxide (H_2_O_2_) is capable of crossing cell membranes and exerts its effects beyond the cell limits [150, 151].

Under physiological conditions, superoxide anion (O_2_^-·^) is the most frequently generated radical. Its main source is the inner mitochondrial membrane during the respiratory chain, particularly the complexes I and III, by inevitable leakage of electrons to O_2_ [152, 153]. O_2_^-·^ can also be formed following electron leakage in a shorter electron transport chain at the endoplasmic reticulum (ER) and during the membrane-bound nicotinamide adenine dinucleotide phosphate oxidase (NOX) activity, which transfers one electron from NADPH to O_2_ [154].

To cope with the continued ROS production, cells have developed antioxidant mechanisms that prevent their accumulation and deleterious actions. Antioxidants, enzymatic or nonenzymatic, can mitigate ROS effects by delaying oxidation or preventing it from happening. In cells, key enzymatic antioxidants are superoxide dismutase (SOD), catalase (CAT), and glutathione peroxidase (GPx) [155], whereas important nonenzymatic antioxidants comprise vitamins C (ascorbic acid) and E (tocopherol), zinc and selenium, glutathione, plant polyphenols, and carotenoids (carotene and *β*-carotene) [156]. Other molecules with moderate antioxidant properties may also be relevant because of their abundance, as is serum albumin [157].

**Figure 3:**
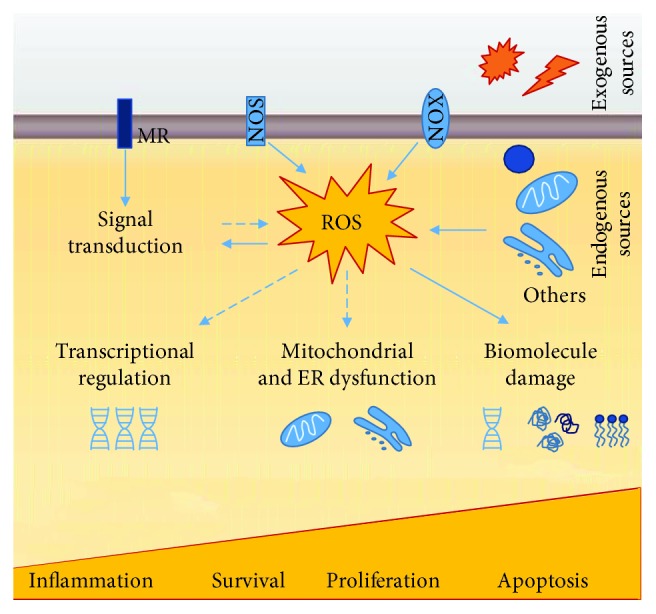
ROS sources and downstream cellular effects. Endogenous sources of ROS include mitochondrial metabolic reactions, NADPH oxidase activity, and microsomal cytochrome P450 detoxification pathways; exogenous sources comprehend ultraviolet radiation, X-rays and gamma-rays, ultrasounds, pesticides, herbicides, and xenobiotics. ROS are normal products of cell metabolism with physiological roles in the organisms. They regulate signalling pathways through changes in the activity of structural proteins, transcription factors, membrane receptors, ion channels, and protein kinases/phosphatases. However, when ROS levels rise, and antioxidant defence cannot neutralize them, the redox homeostasis is disrupted, and a new state referred to as oxidative stress (OS) arises. OS leads to impairment of redox signalling and induces damage to biomolecules. OS has a graded response with minor or moderated changes provoking an adaptive response and homeostasis restauration and violent perturbations leading to pathological insults, damage beyond repair, and even cell death. MR: membrane receptor; NOS: nitric oxide synthase; NOX: NADPH oxidase. Filled arrows indicate a direct action, while dashed arrows indicate indirect or simplified mechanisms.

ROS are normal products of cell metabolism with physiological roles in the organisms. They regulate signalling pathways through changes in the activity of structural proteins, transcription factors, membrane receptors, ion channels, and protein kinases/phosphatases [158] However, when ROS levels rise, and antioxidant defences cannot neutralize them, the redox homeostasis is disrupted, and a new state referred as oxidative stress (OS) arises. OS leads to an impairment of redox signalling and causes molecular damage to biomolecules [159, 160]. OS condition is graded; while minor or moderated changes provoke an adaptive response and homeostasis restauration, higher ones result in violent perturbations that lead to pathological insults, damage beyond repair, and even cell death [159] (Figure 3).

#### 5.1.1. ROS in the Endometrium Cycle

**Figure 4:**
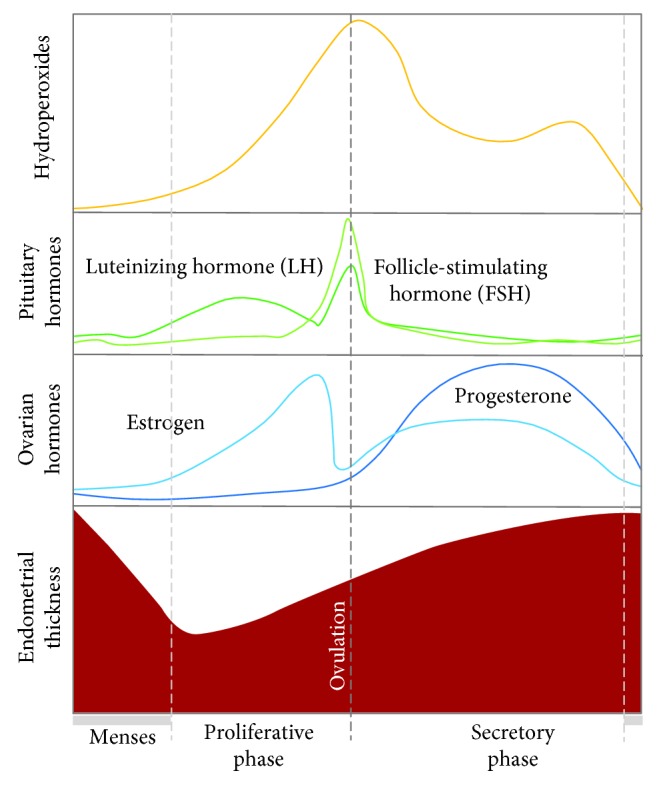
Diagrammatic representation of the different phases of the menstrual cycle, oxidative stress (OS) changes, and fluctuations in ovarian and pituitary hormones. Plasmatic OS marker (hydroperoxides) maximum levels are seen near ovarian and pituitary hormone peaks [161].

ROS are believed to be implicated in the regulation of the endometrial cycle (Figure 4) [161]. NOX-derived O_2_^-·^ has been shown to activate the nuclear factor kappa-light-chain-enhancer of activated B cells (NF-*κ*B) and regulate angiogenesis [162, 163], thus resulting in a determinant role in the endometrial cycle. Variations in SOD, GPx, and lipid peroxides in response to oestrogen and progesterone levels have also been reported [164, 165]. In a late secretory phase, steroid hormone fall reduces SOD activity and, consequently, increases ROS effects [166, 167]. ROS-mediated activation of NF-*κ*B signalling cascade promotes prostaglandin secretion, vasoconstriction, and, ultimately, the endometrial shedding [168–171], at the end of the secretory phase. The exacerbated uterine ROS level and NF-*κ*B activation may result in signalling pathway disruption and in a broad spectrum of uterine-related infertility disorders, as endometriosis [172]. In recurrent pregnancy loss (RPL), increased activity of antioxidant enzymes and decreased markers of OS in endometrial secretions before implantation associated positively with a successful IVF outcome [173]. Moreover, endometrial alterations in progesterone-induced SGK1 (a serine-threonine protein kinase homologous to AKT) were also related to RPL due to impairment in OS defences [174].

#### 5.1.2. ROS and Decidualization

Recent findings suggest that decidual stromal cells evolved from ancestor stromal cell fibroblasts, whose phenotype acquisition is modulated by redox signalling, ER stress, and cellular senescence [175]. In this context, resveratrol, a molecule with antioxidant and anti-inflammatory properties, inhibits decidualization in mice by repression of decidualization markers and abrogation of cellular senescence [176], whereas decidual cell ER sensitive to stressful conditions results in a decrement of decidual functioning [177, 178] and viability [179]. In short, during decidualization, redox-sensitive transcription factors and kinases are activated, making plausible the intervention of ROS and their regulators in this process [180–182] and extending it into placentation. In pregnancy, progesterone stimulates uterine stromal decidualization and decidual SOD expression [183, 184]. In addition, GPx3 is highly expressed in mice decidua, favouring its involvement in uterine transformation and implantation, a point further supported by the reduced pregnancy rates upon GPx3 inhibition [165].

#### 5.1.3. ROS and Regulation of Trophoblast Function

EVTs are also adversely regulated by OS because of their interference with fundamental cellular pathways, reduction of MMP expression, upregulation of proinflammatory cytokine secretion, and induction of mitochondrial dysfunction [185–192]. These consequences disrupt EVT crosstalk within the uterine microenvironment and impair fundamental biological processes as differentiation, proliferation, migration, and vascular remodelling (Table 4). The use of specific antioxidant molecules may have beneficial effects on EVT functions [186, 188, 189].

#### 5.1.4. The Ageing Uterus

In the aged uterus, indirect evidence supports the occurrence of cellular senescence, which is thought to affect decidual transformation [195] and promote preterm births [196, 197]. In addition, reproductively aged mice show age-related increase in uterine NOX and protein carbonylation content, contributing to abnormal decidualization and reduced fertility. NOX inhibition, but not enhanced H_2_O_2_ conversion using a SOD mimetic, restores local redox balance, repairs maternal-foetal interactions, and increases fertility [6]. In line with these results are the recent findings of Banerjee and coworkers reporting that low H_2_O_2_ levels increase EVT invasion, while high levels induce apoptosis [191, 194]. Interestingly, an age-related decrease in adrenal synthesis of dehydroepiandrosterone (DHEA) is believed to grant increased antioxidant capacity to decidualized cells and improve endometrial receptivity [198–200].

On a wider view, either by disturbing uterine decidua or embryo-derived cell functioning, important aspects of modern life style such as obesity, increased maternal age, alcohol consumption, and exposure to substances may act as endocrine disruptors and affect implantation and placentation through OS induction [6, 7, 185, 190, 193, 201].

Therefore, it is now recognized that, at the time of implantation, OS-related alterations in uterine microenvironment lead to a relevant disturbance at the foetus/maternal interface that impairs trophoblast invasion and spiral artery remodelling and stand at the root of major pregnancy-related complications of vascular origin, such as preeclampsia and IUGR.

### 5.2. AGEs, RAGEs, ROS, and Placentation

**Figure 5:**
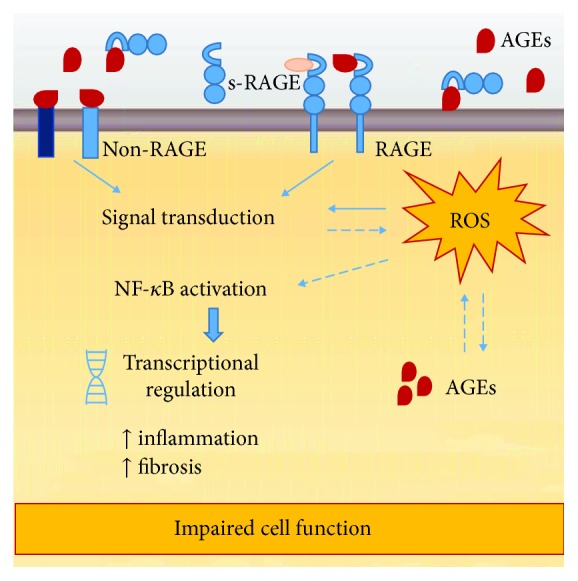
Advanced glycation end-product (AGE) pathological effects. Most of AGE effects are dependent on the interaction AGE/RAGE (receptor of AGE) and the activation of transduction pathways. However, AGEs can bind non-RAGE proteins, and interestingly, RAGE can be activated by other ligands. AGE interactions with membrane receptors trigger various ROS-mediated signalling pathways that converge on NF-*κ*B activation and transcriptional regulation of genes, which impairs cell function. The proteolytic cleavage of extracellular RAGE originates circulating peptides referred as soluble RAGE (sRAGE). It is believed that sRAGEs act as decoy receptors, which scavenge circulating AGEs, preventing them from binding functional membrane RAGE and inducing cellular responses.

Glycation is a nonenzymatic reaction (not to be confused with the enzymatic reaction glycosylation), between reducing sugars (e.g., glucose, fructose, or galactose) and amino groups of proteins, lipids, or nucleic acids. Advanced glycation end-products (AGEs) are the result of a series of glycation reactions [202]. The formation of AGEs was first described by Maillard in the beginning of the 20^th^ century; however, the chemical reactions were only described later in the setting of food research [202]. Briefly, in the classic Maillard reaction, electrophilic carbonyl groups of reducing sugars interact with free amino acid residues (especially arginine or lysine) and form unstable Schiff bases that reverse when glucose levels drop. Further rearrangements result in the formation of the more stable, but still reversible, “Amadori products,” which can react with peptides or protein amino acids, this time irreversibly, leading to the formation of AGEs [203, 204]. The Maillard reaction is not the unique pathway for AGE formation because other reactions involving the formation of carbonyl-containing reactive compounds end up as AGEs [205, 206]. As such, it is not surprising that AGEs are a quite complex, heterogeneous group of compounds, formed either exogenously (e.g., dietary AGEs) and endogenously, by different mechanisms and precursors. ROS, O_2_, and transition metals are catalysers of AGE synthesis [207] and AGE interactions with membrane receptors that trigger various ROS-mediated signalling pathways, such as ERK1/2-MAPK, PI3K-Akt, and p38-SAPK-JNK [208, 209] (Figure 5).

Very little is known about AGE physiological role, and few researches have addressed this issue. Cerami hypothesized that AGEs were protein residues that acted as signals, targeting them to degradation, and that age-related AGE accumulation resulted from loss of efficiency of the removal system [210]. Other authors have explored methylglyoxal, an AGE precursor, as an antimicrobial and anticarcinogenic agent [211].

A handful of papers have also explored AGEs as preconditioners, preparing cells to exacerbated OS, and thus contributing to a future improvement in antioxidant/inflammation response [212–215]. Up to date, nothing is known about a putative antioxidant or antifibrotic effect of dietary AGEs on obstetric-related disorders, although there is a possibility that is worth exploring.

An increase in AGE levels accompanies the ageing process itself and is also a significant contributor and a major risk factor to the development of several age-associated disorders. Higher levels of circulating AGEs or AGE receptor (RAGE) activation have been found in diabetes, hypertension [216], systemic lupus erythematosus [217], rheumatoid arthritis [218], Alzheimer disease [219], and neoplasia [220, 221]. Interestingly, elevated circulation AGEs have also been found in pregnancy-associated complications such as severe preeclampsia [222] and gestational diabetes mellitus (GDM) [223] where it has been positively correlated with proinflammatory markers [224]. In animal models, treatment with soluble RAGE, RAGE inhibitors, and antioxidant molecules ameliorates placental complications [225].

#### 5.2.1. AGEs, ROS, and Regulation of Trophoblast Function

*In vitro* experiments with trophoblasts isolated from first trimester chorionic villi showed that AGE administration increased apoptosis, proinflammatory cytokine production, and monocyte migration. Activation of the NF-*κ*B pathway was crucial to the observed AGE-mediated cell responses, since an inhibitor of this pathway displayed beneficial effects [226]. In accordance, AGEs were found to be upstream molecules that trigger ROS production, activate soluble fms-like tyrosine kinase-1 (sFlt-1), VEGF, and PlGF [227], increase cytokine production in immortalized trophoblast cell lines isolated from first trimester villi (HTR-8/SVneo and Sw.71 cells), and enhance monocyte migration [228, 229]. This inflammatory environment conditions placenta development. Anti-RAGE immunoglobulin or antioxidant treatment also proved effective in reverting AGE-mediated cell effects [227]. Recently, work from Antoniotti et al. showed that uterine AGE levels found in obese women impair uterine transformation and trophoblast function [7].

Overall, data obtained from both *in vivo* and *in vitro* experiments demonstrated that AGEs alter trophoblast function through ROS increase and activation of the NF-*κ*B pathway [227, 229–231], supporting the view that an age-related imbalance in uterine oxidative microenvironment, present even before pregnancy, conditions implantation.

## 6. Concluding Remarks and Future Perspectives

Placenta central function is to supply an adequate amount of blood to properly nourish the foetus. To achieve this purpose, a receptive endometrium is permeated by extravillous trophoblast cells that invade it as deep as the muscular layer. This invasion anchors the placenta to the maternal uterus and guarantees local blood supply through a surprising structural and functional change in maternal spiral arteries: by way of the replacement of their walls by embryo-derived cells, their resistance properties are converted into capacitance features. Such a process requires coordination and cooperation between maternal and foetal tissues.

Similar to key roles played by ROS in processes as oocyte maturation and fertilization, ROS involvement continues in decidualization, implantation, modulation of trophoblast proliferation and differentiation, and embryo development.

A balance between oxidant and antioxidant molecules is vital for a successful ending. The placenta is a growing organ that must evade the adverse effects of homeostasis loss and adapt to reinstall homeostasis. However, when local redox status is significantly disturbed, and severe OS is established, molecular and cellular damage ensues. In the decidualized uterus, those events alter protein function and structure and signalling pathways, disrupt ECM and cytokine production, and hamper the microenvironment at the maternal-foetal interface.

More researchers are convinced that alterations in the foetal-maternal microenvironment before pregnancy, whether by ROS or AGEs, are the culprits and the etiopathogenic roots of pregnancy-related complications of vascular origin. Clearly, we have much to learn, by unravelling ROS-mediated molecular mechanisms dysregulated at the uterus.

## Figures and Tables

**Table 1:** Classification of matrix metalloproteinases.

MMP classification			Enzyme substrates	Cell type/tissue secretion	References
Collagenases	Collagenase-1	MMP-1	Collagen types I, II, III, VII, and X	EVTs, decidua, and uNK	[39–42]
	Collagenase-2	MMP-8	Collagen types I and III	EVTs, decidua	[39, 43, 44]
	Collagenase-3	MMP-13	Collagen type I	EVTs, decidua	[39, 44–46]

Gelatinases	Gelatinase A	MMP-2	Collagen types I, III, IV, V, VII, and X; gelatin; fibronectin; and elastin	EVTs, decidua, and uNK	[39, 42, 44, 47–52]
	Gelatinase B	MMP-9	Collagen types I, III, IV, and V and gelatin	EVTs, decidua, and uNK	[39, 42, 44, 47–50, 52]

Stromelysins	Stromelysin-1	MMP-3	Collagen types III, IV, IX, and X; gelatin; laminin; fibronectin; and elastin	EVTs, decidua	[39, 44, 50, 53]
	Stromelysin-2	MMP-10	Collagen types II, IV, and V; fibronectin; and gelatin	EVTs, decidua, and uNK	[39, 44, 50, 54]
	Stromelysin-3	MMP-11	Collagen type IV	EVTs, decidua, and uNK	[39, 44]

	Matrilysin	MMP-7	Fibronectin and gelatin	EVTs, decidua, and uNK	[39, 41, 42, 44, 45]

	Matrilysin-2	MMP-26	Fibronectin and gelatin	EVTs, decidua	[39, 55, 56]

	Metalloelastase	MMP-12	Elastin and fibronectin	EVTs, decidua, and uNK	[39, 44, 57, 58]

**Table 2:** Molecules secreted in response to decidualization.

Soluble factors	Reference
EGF ↑	[72]
IL-1*β* ↑	[73]
IL-6 ↑	[73]
IL-8 ↑	[73]
IL-10 ↑	[74]
IL-11 ↑	[75, 76]
IL-15 ↑	[76]
IGFBP-1 ↑	[75, 76]
IP-10 ↑	[73]
LIF ↓	[77]
RANTES ↑	[73]
TGF-*β* ↑	[72]
TNF-*α* ↑	[72]
VEGF ↑	[72]

**Table 3:** Soluble factors secretion and its effect on invasion.

	Soluble factor	Secreted by	Effects on trophoblast invasion	References
Proinvasive	CCL14	Decidua	Increase migration by promoting CAM expression alterations (*α*-catenin and integrin *β*5); increase invasion by increasing MMP-12 expression	[64, 78]
	CX3CL1			[78]
	EGF	Decidua and mesenchymal villi	Increase invasion by increasing MMP-9 and TIMP-1 expression	[65, 79–82]
	HGF	Decidua, placental stromal cells, and uNK	Increase invasion by upregulating of H2.0-like homeobox gene	[83, 84]
	IGFBP-1	Decidua	Increase invasion by increasing gelatinolytic activity	[31, 85–87]
	IL-1*β*	Cytotrophoblasts, decidua, macrophages, and uNK	Increase invasion by increasing MMP-2, MMP-9, and urokinase plasminogen activator expression	[78, 88–95]
	IL-6	Cytotrophoblasts and uNK	Increase invasion by increasing MMP-2 and MMP-9 expression	[91, 96–101]
	IL-8	Cytotrophoblasts, decidua, macrophages, and uNK	Increase invasion by increasing MMP-2, MMP-9, uPA, and plasminogen activator inhibitor (PAI) type 1 and 2 expression	[102, 103]
	IL-15	Decidual cells	Increase invasion by increasing MMP-1 expression	[76, 104, 105]
	IP-10	Endometrial stromal cells, uterine glandular cells, and uNK	Increase migration by increasing integrin expression (*α*5 and *β*3)	[106–108]
	LIF	Decidual stromal cells and uNK	Increase adhesion through changes in integrin expression; increase invasion by decreasing TIMP-1 expression	[109–115]
	RANTES	Uterine stromal cells	Increase adhesion and migration by increasing cytolytic activity and integrin expression (*β*1)	[116–118]

	IL-11	Cytotrophoblasts, uNK, and decidua	Involvement in EVT function less understood; inhibiting invasion in HTR-8/SVneo and increasing in JEG-3	[119–121]

Anti-invasive	CXCL14	Decidual stromal cells	Decrease invasion by gelatinase activity suppression	[64]
	IL-10	Macrophages and uNK	Decrease invasion by downregulating MMP-2 and MMP-9 expression	[122]
	INF-*γ*	Cytotrophoblasts, decidua, and uNK	Decrease invasion by decreasing insulin-like growth factor receptor-II	[123–127]
	Kisspeptin-10	Cytotrophoblasts and decidua	Decrease invasion by binding to g protein-coupled receptor kisspeptin-1 receptor increasing Ca^2+^ intracellular levels	[123–127]
	TGF-*β*	Cytotrophoblasts, decidua, and uNK	Decrease invasion by increasing of TIMP-1 and TIMP-2 and plasminogen activator inhibitor type 1 and 2 expression; increases adhesion by upregulating the expression of CAM (ezrin and e-cadherin)	[62, 79, 85, 123–125, 128–130]
	TNF-*α*	Cytotrophoblasts, decidua, macrophages, and uNK	Decrease invasion by upregulation plasminogen activator inhibitor type 1 expression	[123, 125, 130–132]
	VEGF	Decidua, macrophages, and uNK	Decrease invasion by inhibiting urokinase plasminogen activator expression	[133]

**Table 4:** ROS-mediated regulation of trophoblast function.

Agent	Molecular effects	EVT functions	Reference
Decanoic acid	Disrupts mitochondrial function **↑** ROS generation ↓ Akt and ERK1/2 pathways	↓ proliferation ↓ invasion	[185]
Trichloroethylene	Disrupts mitochondrial function **↑** ROS generation **↑** proinflammatory cytokine production	—	[190]
Benzo(a)pyren-7,8-dihydrodiol-9,10-epoxide	Disrupts mitochondrial function ↑ ROS generation ↓ SOD activity Induces apoptosis	↓ invasion	[193]
Higher H_2_O_2_ concentrations	Induces apoptosis	↓ invasion	[194]

Lower H_2_O_2_ concentrations	↑ STAT 1 and 3 pathways ↑ MMP-9/TIMP-1 ratio	↑ invasion	[191]
Selenium (under hypoxic conditions)	↓ mitochondrial stress	↑ proliferation ↑ migration	[186]
Edaravone (under hypoxic conditions)	↓ ROS production	↑ proliferation ↑ migration ↑ invasion	[187]
Flavonoids (under hypoxia/reoxygenation)	↓ ROS production	—	[189]
